# Supplementary material for: Photothermal Thin Films with Highly Efficient NIR Conversion for Miniaturized Liquid-Crystal Elastomer Actuators
Source: Polymers (Basel). 2022 Jul 24;14(15):2997. doi: 10.3390/polym14152997 (PMC9330919; doi:10.3390/polym14152997)
Supplement: Supplementary file 1 [file polymers-14-02997-s001.zip › polymers-1811397-supplementary.pdf]

polymers

MDPI

Supplementary Materials

1. Operational principles of the LCE crawling device

This document describes the details of the operational principles of the LCE crawling device [11]. The device mimics the rectilinear locomotion of snakes by utilizing the characteristics of reversible shape shifting of the arched actuator caused by the LC-to-isotropic phase transition. Figure S1(a) shows the crawler at its original stable state. Under NIR illumination, the PTF of the arched actuator absorbs light energy and heats up the arched LCE structure (Figure S1(b)). As the temperature is above the LC-to-isotropic transition temperature, the actuator extends into a shape that is almost flat; thus, the front leg slides forward and the rear leg slides backward, as illustrated in Figure S1(c). At this moment, the device almost stays in its original position. As NIR illumination is terminated, the actuator temperature decreases gradually, and the actuator starts to retreat to its original shape. Concurrently, due to the asymmetrical two-leg design, the edge of the front leg hooks on the ground so that the body of the crawler is pulled forward while the round surface of the rear leg slides on the ground alongside, as shown in Figure S1(d). The completion of an actuation cycle is shown in Figure S1(e) as the crawler returns to its original shape.

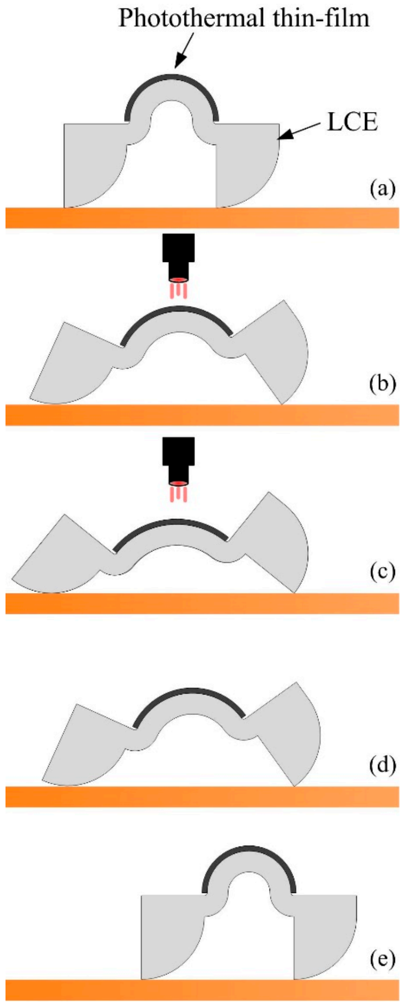

Figure S1. The operational principles of the actuation scheme using the proposed PTF.

2. Technique for extracting the curvature of the arched actuator

The snapshots (digital photographs) of the crawler locomotion are taken by using a digital camera (Panasonic DMC-GF3) which points perpendicular to the side surface of the device. Then, the curvature of the actuator for each snapshot can be calculated by using an image processor software Image J. Figure S2 illustrates the definition of the curvature, which is the reciprocal of the radius of the dotted red circle enclosing the outer surface of the PTF. When using the Image J, the edge of the outer surface of the PTF is manually marked by 10 points or more using a mouse. Then, the software calculates a best-fit circle based on these points, and the curvature of the circle can be estimated.

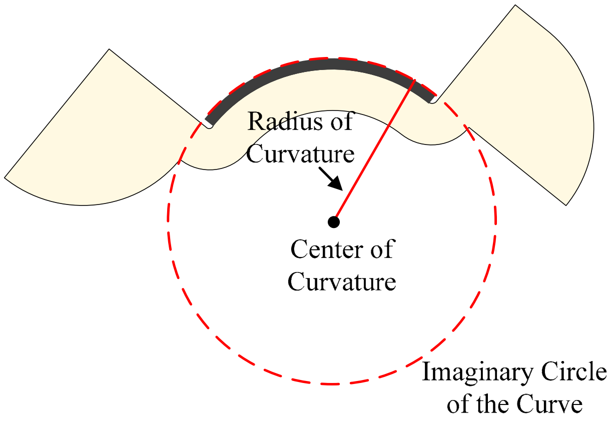

Figure S2: Schematic diagram of the radius of curvature.
